# Ameloblastic fibroma: A rare case appearing as a mixed radiographic image

**DOI:** 10.4317/jced.51471

**Published:** 2014-12-01

**Authors:** Jurema-Freire-Lisboa de Castro, Andreza-Veruska-Lira Correia, Lucas-Alexandre-Moraes Santos, Luiz-Antônio-Portela Guerra, Flávia-Maria-de-Moraes Ramos-Perez, Danyel-Elias-da Cruz Perez

**Affiliations:** 1 DDS, PhD. Department of Clinical and Preventive Dentistry, Federal University of Pernambuco, Recife, Pernambuco, Brazil; 2 DDS, MSc. Department of Clinical and Preventive Dentistry, Federal University of Pernambuco, Recife, Pernambuco, Brazil; 3 DDS. Department of Oral and Maxillofacial Surgery, Hospital da Restauração, Recife, Pernambuco, Brazil; 4 DDS, PhD. Department of Oral and Maxillofacial Surgery, Hospital Geral de Areias, Recife, Pernambuco, Brazil

## Abstract

Ameloblastic fibroma (AF) is a benign tumor of mixed odontogenic origin, which affects predominantly young individuals. AF appearing as a mixed radiographic image is very rare. This report describes a case of AF in a 12-year-old male identified during a routine radiographic exam for orthodontic treatment planning. The panoramic radiography revealed a well-defined multilocular mixed image located in the mandible between the roots of the left mandibular second premolar and first molar. The lesion was excised under local anesthesia. Histopathological analysis revealed islands of epithelial cells and columnar peripheral cells showing a nucleus in inverted polarization, interspersed with spindle-shaped cells and abundant extracellular matrix deposition. No atypia was observed. The diagnosis of AF was established. No tumor recurred up to 30 months after treatment. Although rare, AF should be also considered in the differential diagnosis of mixed radiographic images of the jaws in young patients.

** Key words:**Ameloblastic fibroma, differential diagnosis, incidental finding, mixed image, radiographic features.

## Introduction

Ameloblastic fibroma [AF] is a rare tumor, accounting for 2% of all odontogenic tumors, characterized by simultaneous neoplastic proliferation of mesenchymal and epithelial components, with no formation of hard dental tissues (1). The tumor predominantly affects the posterior region of the mandible (2-4) in patients during the first and second decades life (1-3).

AF exhibits slow growth and most cases present with a painless swelling or are discovered during routine radiographic exams (1-5). Delays or alterations in the sequence of tooth eruption are also important findings, as 75% of the cases are associated with an impacted tooth (1-3). Radiographically, AF appears as a well-defined, unilocular or multilocular radiolucent lesion, with sclerotic radiopaque margins (3,4). AF may be initially identified in routine radiographic exams (4).

Microscopically, the lesion shows cords and islands of neoplastic odontogenic epithelium interspersed by neoplastic mesenchymal tissue that resembles the dental papilla. Microscopic differential diagnoses include odontogenic myxoma and odontogenic fibroma (6). Although it is rare, malignant transformation may occur, mainly in recurrent tumors (7,8). As no hard tissue is observed, AF appearing as a mixed radiographic image is very unusual (4). Thus, this report describes a case of AF appearing as mixed radiographic image, which was identified as incidental finding. In addition, clinical and radiographic features are discussed as well as differential diagnosis and treatment.

## Case Report

A 12-year-old male was referred for evaluation of a lesion in the left body of the mandible identified in a radiographic exam for orthodontic treatment planning. The medical history revealed no prior trauma or episodes of pain in the affected region. The extraoral examination revealed a painless slight swelling in the inferior portion of the face, corresponding to the region of the left mandibular second premolar and first molar. The lesion presented a firm consistency and no signs of inflammation were observed. On the intraoral examination, the same features were observed, with the discrete swelling showing a hard consistency and recovered by mucosa of normal appearance. There were no carious or periodontal lesions in the left mandibular teeth. However, a diastema was found between the left mandibular second premolar and first molar.

**Figure 1:**
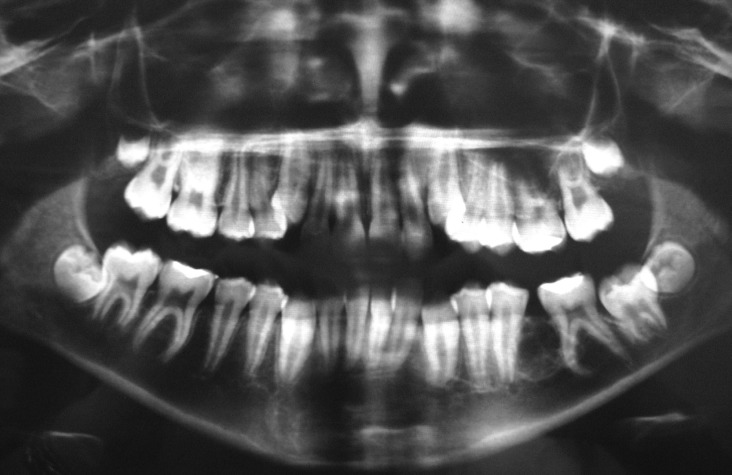
A well-defined multilocular mixed image, with sclerotic borders, located between the roots of the left mandibular second premolar and first molar.

The panoramic radiography revealed a well-defined multilocular mixed image, with sclerotic borders, located in the mandible, laterally and between the roots of the left mandibular second premolar and first molar, measuring approximately 1.5 cm in extension. Thin septa were observed within lesion. The radiopaque portion was restricted to the peripheral area of the lesion. Moreover, divergence of the roots was also observed (Fig. 1). Ossifying fibroma, extra-follicular variant of adenomatoid odontogenic tumor, calcifying cystic odontogenic tumor, calcifying epithelial odontogenic tumor, and myxoma were the main hypotheses of diagnosis.

**Figure 2:**
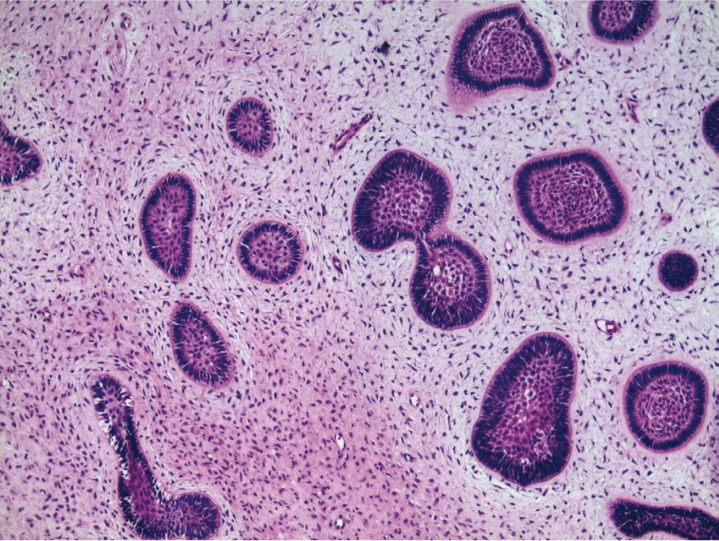
Several spindle-shaped cells and abundant extracellular matrix deposition were found between the islands of neoplastic cells (HE, x100).

**Figure 3:**
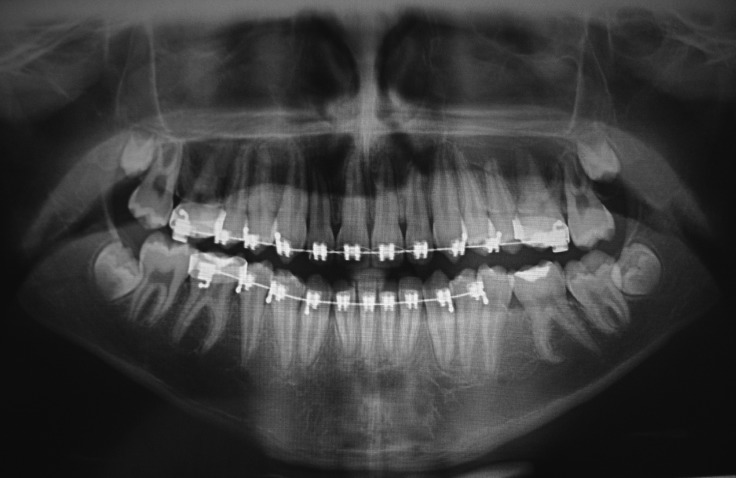
No signs of tumor recurrence after 12 months of the treatment.

As the lesion was well circumscribed, under local anesthesia, the patient was submitted to excision of the lesion. Enucleation of the tumor was performed, followed by curettage of the surrounding bone. The surgical specimen was fixed in formalin and sent for histopathological analysis, which revealed an odontogenic tumor formed by islands of epithelial cells, with the columnar peripheral cells showing nucleus in inverted polarization. Numerous spindle-shaped cells and abundant extracellular matrix deposition were found between the islands of neoplastic cells (Fig. 2). No atypia was observed. Thus, the diagnosis of AF was established. The patient is currently under periodical clinical and radiographic follow up, with no signs of tumor recurrence 30 months after treatment. In addition, orthodontic treatment was initiated (Fig. 3).

## Discussion

AF predominantly affects young patients, with a mean age of 15 years (2-4). However, an extensive literature review found that approximately 25% of cases occur in patients over 22 years of age, likely due to the limited access to routine dental treatment in the population studied (4).

Although few cases have been reported, the majority of studies indicate a slight predilection for the male gender (2-4). The posterior region of the mandible is the most affected site (3-5), but large tumors may expand to anterior regions of the mandible. Unlike the present case, AF is very often associated with an impacted tooth (1), which may lead to delayed tooth eruption. The tumor exhibits insidious growth and an increase in volume is the most frequent clinical finding (4). However, the presence of ulceration and pain does not exclude the diagnosis of AF, as these symptoms have been reported in an extensive review carried out by Chen *et al.* (4). On the other hand, about 20% of the tumors are diagnosed just after routine dental radiography (4), as occurred in this case. Additionally, in the current case, the tumor was located between the roots of the left mandibular second premolar and first molar, causing a diastema between these teeth.

Radiographically, AF appears as a well-defined unilocular or multilocular radiolucent image with a mean size of 4.0 cm (4). Ameloblastoma, odontogenic myxoma, keratocystic odontogenic tumor and central giant cell lesion should be considered in the differential diagnosis (3,9). In the present report, the presence of thin septa producing a stepladder pattern resembled that observed in myxoma, although odontogenic myxoma occurs most commonly in patients during third and fourth decades of life (10). In addition, odontogenic myxoma may present diffuse calcifications (11). Despite the asymptomatic cases of AF usually appear as an unilocular radiolucency (4), the present case was characterized as multilocular mixed image. The occurrence of similar cases of AF is very rare (4).

Particularly in this case, mixed radiographic lesions, such as ossifying fibroma, extra-follicular variant of adenomatoid odontogenic tumor, calcifying cystic odontogenic tumor and calcifying epithelial odontogenic tumor were also considered as hypotheses of diagnosis (12-14). However, except for adenomatoid odontogenic tumor, these tumors are uncommon in this age group (13). The radiopaque foci in these mixed tumors usually are cen-trally located or distributed along entire lesion. On the other hand, in this case, the radiopaque area was peripherically located. The lesion probably induced a peripheral bone neo-formation, resulting in a mixed radiographic image. Moreover, the more radiopaque area may represent a region with less bone resorption when compared to remaining radiolucent area. Despite the radiopaque foci, no hard tissues were observed at microscopical analysis.

Because of AF is often associated with non-erupted teeth, it may initially be interpreted as a dentigerous cyst (1,2). Besides AF, other lesions can be identified as an incidental radiographic finding (9), as occurred in the present case. This underscores the inestimable value of access to periodic dental exams, especially radiographic exams among children and adolescents.

Microscopically, AF is characterized by the proliferation of odontogenic epithelium immersed in a mixoid cellularized tissue similar to a dental papilla with different degrees of inductive changes and no formation of hard dental tissue (1,5). The epithelial component is characterized by the proliferation of islands, strings or projections of odontogenic epithelium and a peripheral layer of cuboid or columnar cells with central areas resembling the stellate reticulum of the dental organ (1,4). The amount and density of the epithelial component may vary from region to region within the same tumor. Cystic degeneration and mitotic activity are uncommon findings (1,3). Histopathological differential diagnoses include odontogenic myxoma and odontogenic fibroma. In contrast to current case, the presence of epithelial islands is an infrequent finding in odontogenic myxoma (15). Although the epithelium-poor odontogenic fibroma present a loose connective tissue, with a fibromyxoid appearance in some areas, the epithelial islands are inactive and scarce (6). The epithelium-rich odontogenic fibroma shows a variable amount of inactive odontogenic epithelial islands (6), whereas the ameloblastic fibroma presents islands and strings of neoplastic epithelial cells, as occurred in this case. Moreover, the epithelial islands observed in epithelium-rich odontogenic fibroma are immersed in a cellular fibroblastic connective tissue (6).

While the malignant transformation of AF is rare, attention should be paid to the occurrence of mitosis, which is a finding to consider the possibility of an ameloblastic fibrosarcoma (7). Mesenchymal proliferation in the interior of the tumor resulting in loss of the epithelial component should also be considered an important event, as it may indicate a modification seen in the malignant transformation of an AF (4). However, the diagnosis of malignant transformation is only established from the retrieval of the primary tumor or recurring tumors in which the original histological features of the AF can be observed (1).

The appropriate treatment for AF remains matter of debate. A conservative approach, with excision followed by curettage, as performed in the present case, appears to be the most adequate treatment (3). However, large tumors or cases with multiple recurrences may require more extensive resections (1). Recently, an extensive review reported a recurrence rate of 16.3%, with malignant transformation to ameloblastic fibrosarcoma occurring in 6.4% of the reviewed cases (5). These findings underscore the need for a complete excision of the tumor as well as long-term clinical and radiographic follow up, with special attention given to cases of recurrences, which have a greater risk of malignant transformation (4). Nevertheless, approximately two thirds of the ameloblastic fibrosarcomas representing malignant tumors de novo (8).

In summary, AF is a rare tumor and presents a good prognosis. However, patients must be followed up carefully after treatment and special care should be given to recurring tumors. Radiographic exams should always be requested to complement the periodic clinical exam because several mandibular lesions may present as an incidental radiographic finding. Although rare, AF should be considered in the differential diagnosis of mixed lesions of the jaws in patients during childhood and adolescence.
